# Association of *KDR* (rs2071559, rs1870377), *CFH* (rs1061170, rs1410996) genes variants and serum levels with pituitary adenoma

**DOI:** 10.1002/mgg3.2289

**Published:** 2023-10-06

**Authors:** Akvile Bruzaite, Greta Gedvilaite, Loresa Kriauciuniene, Rasa Liutkeviciene

**Affiliations:** ^1^ Ophthalmology Laboratory Neuroscience Institute, Lithuanian University of Health Sciences, Medical Academy Kaunas Lithuania

**Keywords:** *CFH*, hormonal activity, invasiveness, *KDR*, pituitary adenoma, recurrence, rs1061170, rs1410996, rs1870377, rs2071559, serum levels

## Abstract

**Introduction:**

Pituitary adenomas (PA) are slow‐growing, benign tumors that usually do not metastasize to other body organs. Although they are referred to as benign, tumor growth can eventually put pressure on nearby structures, spread to surrounding tissues, and cause symptoms. The exact cause of PA is unknown, and the pathogenesis is multifactorial.

**Methods:**

Our study included PA patients and healthy volunteers. Genomic DNA was extracted using the DNA salting‐out method. All participants were genotyped for the *KDR* rs2071559, rs1870377, *CFH* rs1061170, and rs1410996 polymorphisms. Serum levels of KDR and CFH were examined using the ELISA method.

**Results:**

The results of the present study showed that *KDR* rs2071559 A allele was associated with the occurrence of PA, hormonally active PA, invasive PA, and PA without recurrence development. *KDR* rs1870377 increased the probability of invasive PA and PA recurrence. *CFH* rs1061170 C allele was associated with hormonally active PA and the T allele was associated with non‐invasive PA development.

**Conclusion:**

*KDR* rs2071559, rs1870377, and *CFH* rs1061170 could be potential biomarkers associated with PA.

## 1 INTRODUCTION

Pituitary adenomas (PA) are slow‐growing benign tumors that usually do not metastasize to other body organs (Javanbakht et al., 2018; Russ et al., 2022). Although called benign, tumor growth can eventually pressure nearby structures, spread to surrounding tissues, and cause symptoms due to the metabolic consequences of excess hormone secretion (Larkin et al., 2000; Møller et al., 2019). Patients with PA may initially experience neurological symptoms: headaches, changes in visual function, or endocrine disorders such as infertility, decreased libido, and galactorrhea (Lake et al., 2013). PA accounts for about 10%–25% of all intracranial tumors, and the occurrence of clinically diagnosed PA is 1 per 1000 cases in the general population (Daly & Beckers, 2020; Yang & Li, 2019). Adenomas are classified according to size, location, cell type, neoplastic behavior, and hormonal activity (Ho et al., 2021; Holmes et al., 2007; Ken et al., 2021). Clinically active PAs (prolactinomas, somatotroph, corticotroph, thyrotrophin, and gonadotroph adenomas) make excess hormones, whereas non‐functioning tumors do not show signs and symptoms of hypersecretion (Mehta & Lonser, 2017; Trouillas et al., 2020; Varlamov et al., 2019). The exact cause of PA is unknown, and its pathogenesis is multifactorial. Genetic predisposition implies the existence of germline DNA mutations that have a variety of impacts on pituitary cellular biology (Jaffrain‐Rea et al., 2011).

Abnormal gene expression is linked to genetic changes such as gene amplification or allele loss and epigenetic changes such as promoter methylation (Vandeva et al., 2010). While thousands of genetic variants, including single nucleotide polymorphisms (SNPs), are related to different types of cancer, the molecular mechanisms of the diseases are still not fully understood (Bakhtiari et al., 2020). SNP analysis is a valuable predictor of cancer risk in particular populations where this polymorphism is frequently detected (Deng et al., 2017). More data on PA factors are needed to make it easier to predict tumor occurrence, formation, and invasiveness. New molecular markers can optimize treatment and increase patients' survival (Alwi, 2005).

Vascular endothelial growth factors (VEGFs) and receptors (VEGFRs) control angiogenesis – the later creation of blood vessels from pre‐existing vessels, and vasculogenesis – the early embryonic development of blood vessels from precursor cells. VEGF‐A and its receptors VEGFR‐1 (Flt‐1) and VEGFR‐2 (KDR/Flk‐1) play major roles in physiological as well as pathological tumor angiogenesis (Shibuya, 2011). VEGFR2/KDR is more widely distributed and expressed in all blood vessels at endothelial contact sites and, therefore, could be found in tumor vasculature. Kinase insert domain receptor (KDR) is widely regarded as having a crucial part in mediating VEGF‐induced responses in angiogenesis (Rydén et al., 2003; Fu et al., 2014). *KDR* gene mutations, insertions, and deletions are associated with cancer occurrence, and alterations are observed in 3.02% of all cancers including lung adenocarcinoma, melanoma, colon adenocarcinoma, and conventional glioblastoma (The AACR Project GENIE Consortium, 2017).

The complement system is a component of innate immunity that plays diverse roles in the inflammatory response, hemostasis, and embryogenesis and is implicated in various stages of tumorigenesis and cancer progression (Afshar Kharghan, 2017). Complement factor H (CFH) is one of the key regulators in the alternative complement pathway, which has been known to inhibit the complement pathway by binding to C3b and destroying the C3 convertase (Ezzeldin et al., 2015). Changes in the *CFH* gene are associated with lung cancer (Yoon et al., 2019). Therefore, this research aims to identify SNPs of *KDR* rs2071559, rs1870377, and *CFH* rs1061170, rs1410996 as well as KDR and CFH serum levels in PA patients and healthy subjects and relate the obtained results to PA hormonal activity, invasiveness, and recurrence.

## 2 MATERIALS AND METHODS

### 2.1 Patients and ethical requirements

Permission (No. BE‐2‐47; approved date: 25 December 2016) to perform the research was approved by the Ethics Committee for Biomedical Research at the Lithuanian University of Health Sciences (LUHS). All subjects have signed an agreement according to the Declaration of Helsinki. The study was conducted at the Laboratory of Ophthalmology, Neuroscience Institute, LUHS. Study subjects were divided into two groups:

- Group I: patients with PA (*N* = 100) comprised 39 men and 61 women. Inclusion criteria for the study were: adult (age ≥18 years); PA diagnosed and confirmed by magnetic resonance imaging (MRI); suitable patients' general health and absence of other tumors. Moreover, PA patients were distributed into subgroups by invasiveness, hormonal activity, and recurrence. Invasiveness, hormonal activeness, and recurrence evaluation were described previously in our studies (Glebauskiene et al., 2017; Sidaraite et al., 2020; Yoon et al., 2019).
- Group II: healthy control group comprised 112 women and 88 men, with good general health on the examination date.

### 2.2 SNP selection

The *KDR* gene is located on the long arm of chromosome 4 (4q12), and the *CFH* gene is located on the long arm of chromosome 1 (1q31.3) (https://www.ncbi.nlm.nih.gov/gene/). For our study, we selected SNPs of the *KDR* gene: rs2071559 (in the promoter region, substitution 604A>G, NC_000004.11:g.55992366A>G (https://www.ncbi.nlm.nih.gov/snp/rs2071559/#hgvs_tab)) and rs1870377 (in exon 11, substitution 1719A>T, NC_000004.11:g.55972974T>A (https://www.ncbi.nlm.nih.gov/clinvar/variation/134603/)) (Al Awaida et al., 2021; Hu et al., 2017). From the gene *CFH*, we selected two additional SNPs: rs1061170 (in exon 9, substitution 1277C>T, NC_000001.10:g.196659237C>T (https://www.ncbi.nlm.nih.gov/clinvar/variation/294490/)) and rs1410996 (in intron 14, substitution 543A>G, NC_000001.10:g.196696933G>A (https://www.ncbi.nlm.nih.gov/clinvar/variation/16557/)) (Al Awaida et al., 2021; Neto et al., 2021; Suchankova et al., 2009). Increased VEGF/KDR signaling promotes angiogenesis and plays a key role in chronic inflammation (Paradowska‐Gorycka et al., 2019). The gene *CFH* is involved in the removal of waste and damaged cells from the body. It is suggested that deficiency of CFH and accumulation of waste and damaged cells in the body may lead to oxidative stress and inflammation (He & Karin, 2011; Armento et al., 2021).

Since both *KDR* and *CFH* genes are associated with inflammation, they may also be linked to cancer development and progression (Singh et al., 2019). Because cancer cells have a relatively high metabolic demand for oxygen and nutrients to continue growing, angiogenesis is important for tumor development. The tumor vasculature has been found to express KDR at higher levels than the normal vasculature, and it has been hypothesized that inhibition of angiogenesis may lead to tumor growth arrest (Holmes et al., 2007). CFH is likely involved in the resolution of inflammation in lipid‐rich deposits that have accumulated in the arteries, brain, eyes, and kidneys (Meri & Haapasalo, 2020). This accumulation can activate the complement system, which triggers the development of an inflammatory phenotype in microglia, and phagocytic cells in the brain (Shen et al., 2003). Therefore, poor complement regulation may be involved in carcinogenesis, and complement activation may also interfere with tumor clearance by the immune system (Laskowski et al., 2020; Reis et al., 2018).

The pathogenesis of diseases associated with variations in the *KDR* and *CFH* genes involves multiple complex pathways and mechanisms, and further research is needed to fully understand the underlying processes of PA.

### 2.3 DNA extraction and genotyping

Blood for DNA extraction was collected in EDTA tubes. Genomic DNA was extracted using the DNA salting‐out method from 200 μL peripheral blood (white blood cells). The concentrations and purity indexes of DNA in each blood sample were evaluated by UV spectrophotometry (Agilent Technologies, Cary 60 UV– Vis) as the ratio absorbance 260/280 nm. All samples presented a purity index between 1.8 and 2.0. All participants were genotyped for *KDR* gene rs2071559, rs1870377, *CFH* gene rs1061170, and rs1410996 polymorphisms at the Laboratory of Ophthalmology, LUHS. SNPs were determined using TaqMan® genotyping assays (Applied Biosystems; Thermo Fisher Scientific), C__15869271_10 (rs2071559), C__11895315_20 (rs1870377), C___8355565_10 (rs1061170), C___2530294_10 (rs1410996). The genotyping was conducted using the real‐time polymerase chain reaction (RT‐PCR) method according to the manufacturer's recommendations using a Step One Plus RT‐PCR system (Applied Biosystems).

### 2.4 ELISA

For serum preparation, peripheral venous blood was collected. The blood samples were incubated for 30 min at room temperature and centrifuged. The serum was removed from the pellet, transferred into 2 mL tubes, frozen, and stored at −80°C until analysis. Following the manufacturer's instructions, serum KDR levels were evaluated in PA patients and control subjects. The assay was performed using an Aviva Systems Biology KDR ELISA Kit (Human) based on standard sandwich ELISA technology, with a sensitivity of <0.236 ng/mL. Also, serum CFH levels were determined following the manufacturer's instructions in PA patients and control subjects, using a sandwich‐type Invitrogen Complement Factor H ELISA Kit (Human), with a sensitivity of 2 ng/mL.

### 2.5 Statistical analysis

Statistical data analysis was performed using SPSS/W 27.0 software (Statistical Package for the Social Sciences for Windows, Inc.) statistical data analysis program. The genotype distribution of SNPs was analyzed using the Chi‐square test. To estimate the association between *KDR* rs2071559, rs1870377, *CFH* rs1061170, and rs1410996 and PA development, odds ratios (ORs) and 95% confidence intervals (CIs) were computed using binary logistic regression analysis. A two‐sided *p* < 0.05 was considered to be statistically significant. The Akaike information criterion (AIC) selected the best genetic model. Statistically significant differences were considered when *p* < 0.05. Genotypes were assessed using indicator variables with the common homozygote as a reference. The serum KDR and CFH levels were examined in the groups of PA patients and healthy individuals using the Mann–Whitney *U* test. Also, we performed haplotype association analysis in PA and control groups. It was performed using the online SNPStats website (https://www.snpstats.net/snpstats/). Linkage disequilibrium (LD) was measured using D′ and *r* ^2^. The associations between the haplotypes and PA were calculated using logistic regression. The associations were introduced as ORs and 95% CI and measures in PA and healthy subjects.

## 3 RESULTS

A case–control study was conducted involving 300 individuals who were divided into two groups: the control group (*N* = 200) and a group of subjects with pituitary adenoma (*N* = 100). After forming the groups of subjects' an analysis of single nucleotide polymorphisms of *KDR* rs2071559, rs1870377, and *CFH* rs1061170, rs1410996 was performed. The PA group consisted of 100 persons: 39 men (39.0%) and 61 women (61.0%). The average age of patients was 52.4 years. The patients' group was later divided into subgroups by PA's hormonal activity, invasiveness, and recurrence. The control group consisted of 200 individuals: 88 men (44.0%) and 112 women (56%). The average age of the control group was 69.1 years. Further analysis was adjusted by age after considering the statistically significant age difference in the comparison control and PA groups. The demographic data of the subjects are presented in Table 1.

**TABLE 1:** Demographic characteristics of study subjects.

Characteristics	Group		*p*‐value
	PA (*N* = 100)	Control group (*N =* 200)	
Male, *n* (%)	39 (39.0)	88 (44.0)	0.409^a^
Female, *n* (%)	61 (61.0)	112 (56.0)	
Age years; mean (SD)	52.4 (13.5)	69.1 (5.2)	0.001^b^
Invasiveness, *n* (%)	64 (64.0)		
Hormonal activity, *n* (%)	55 (55.0)		
Recurrence, *n* (%)	26 (26.0)		

Abbreviations: PA, pituitary adenoma; SD, standard deviation; *p*‐value – significance level (differences considered significant when *p* < 0.05).

^a^ Pearson Chi‐Square.

^b^ Student's *t* test.

### 3.1 *KDR* rs2071559, rs1870377, and *CFH* rs1061170, rs1410996 associations with PA occurrence

After analyzing the genotypes and alleles of *KDR* rs2071559, rs1870377, and *CFH* rs1061170, rs1410996, it was found that only the *KDR* rs2071559 AA genotype and A allele were statistically significantly more frequent in PA patients compared to the control group individuals (39.0 vs. 26.5, *p* = 0.027, 60.5 vs. 51.0, *p* = 0.028) (Table 2). There were no statistically significant differences between *KDR* rs1870377, *CFH* rs1061170, and rs1410996 distribution of genotypes and alleles for patients with PA and the control group.

**TABLE 2:** Distribution of genotypes and alleles of *KDR* rs2071559, rs1870377 and *CFH* rs1061170, rs1410996 polymorphisms in patients with PA and control group.

Polymorphism	PA, *N* (%)	Control group, *N* (%)	*p*‐value
*KDR* rs2071559			
AA	39 (39.0)^a^	53 (26.5)^a^	0.075
AG	43 (43.0)	98 (49.0)	
GG	18 (18.0)	49 (24.5)	
Total	100 (100)	200 (100)	
Allele			
A	121 (60.5)	204 (51.0)	**0.028**
G	79 (39.5)	196 (49.0)	
*KDR* rs1870377			
TT	53 (53.0)	108 (54.0)	0.445
TA	37 (37.0)	80 (40.0)	
AA	10 (10.0)	12 (6.0)	
Total	100 (100)	200 (100)	
Allele			
T	143 (71.5)	296 (74.0)	0.515
A	57 (28.5)	104 (26.0)	
*CFH* rs1061170			
TT	43 (43.0)	72 (36.0)	0.180
TC	49 (49.0)	98 (49.0)	
CC	8 (8.0)	30 (15.0)	
Total	100 (100)	200 (100)	
Allele			
T	135 (67.5)	242 (60.5)	0.944
C	65 (32.5)	158 (39.5)	
*CFH* rs1410996			
GG	33 (33.0)	72 (36.0)	0.699
GA	57 (57.0)	104 (52.0)	
AA	10 (10.0)	24 (12.0)	
Total	100 (100)	200 (100)	
Allele			
G	123 (61.5)	248 (62.0)	0.905
A	77 (38.5)	152 38.0)	

Abbreviations: PA, pituitary adenoma; *p*‐value, significance level (differences considered significant when *p* < 0.05).

*Note:* Significant results are indicated in bold.

^a^ AA vs. AG+GG *p* = 0.027.

Binominal logistic regression analysis of *KDR* rs2071559, rs1870377, and *CFH* rs1061170, rs1410996 showed no statistically significant differences between PA and control group (Table S1).

### 3.2 *KDR* rs2071559, rs1870377, and *CFH* rs1061170, rs1410996 associations with PA hormonal activity

After evaluating the distribution of genotypes and alleles of *KDR* rs2071559, rs1870377, and *CFH* rs1061170, rs1410996 in hormonal active PA and the control groups, it was found that both *KDR* rs2071559 AA genotype and A allele were statistically significantly more frequent in hormonal active PA group compared to a control group (41.8 vs. 26.5, *p* = 0.028, 62.7 vs. 51.0, *p* = 0.029, respectively). Also, *CFH* rs1061170 CC genotype and C allele were statistically significantly less frequent in the hormonal active PA group than in the control subjects (3.6 vs. 15.0, *p* = 0.024, 28.2 vs. 39.5, *p* = 0.030, respectively). Analysis of *KDR* rs1870377 and *CFH* rs1410996 polymorphisms revealed no statistically significant differences between hormonal active PA and the control groups (Table 3).

**TABLE 3:** Distribution of genotypes and alleles of *KDR* rs2071559, rs1870377, and *CFH* rs1061170, rs1410996 polymorphisms in active PA patients and the control group.

Polymorphism	PA with hormonal activity, *N* (%)	Control group, *N* (%)	*p*‐value
*KDR* rs2071559			
AA	23 (41.8)^a^	53 (26.5)^a^	0.077
AG	23 (41.8)	98 (49.0)	
GG	9 (16.4)	49 (24.5)	
Total	55 (100)	200 (100)	
Allele			
A	69 (62.7)	204 (51.0)	**0.029**
G	41 (37.3)	196 (49.0)	
*KDR* rs1870377			
TT	33 (60.0)	108 (54.0)	0.611
TA	18 (32.7)	80 (40.0)	
AA	4 (7.3)	12 (6.0)	
Total	55 (100)	200 (100)	
Allele			
T	84 (76.4)	296 (74.0)	0.614
A	26 (23.6)	104 (26.0)	
*CFH* rs1061170			
TT	26 (47.3)	72 (36.0)	0.053
TC	27 (49.1)	98 (49.0)	
CC	2 (3.6)^b^	30 (15.0)^b^	
Total	55 (100)	200 (100)	
Allele			
T	79 (71.8)	242 (60.5)	**0.030**
C	31 (28.2)	158 (39.5)	
*CFH* rs1410996			
GG	16 (29.1)	72 (36.0)	0.430
GA	34 (61.8)	104 (52.0)	
AA	5 (9.1)	24 (12.0)	
Total	55 (100)	200 (100)	
Allele			
G	66 (60.0)	248 (62.0)	0.703
A	44 (40.0)	152 (38.0)	

Abbreviations: PA, pituitary adenoma; *p*‐value, significance level (differences considered significant when *p* < 0.05).

*Note:* Significant results are indicated in bold.

^a^ AA vs. AG+GG *p* = 0.028.

^b^ CC vs. TT+TC *p* = 0.024.

We analyzed *KDR* rs2071559, rs1870377, and *CFH* rs1061170, rs1410996 associations with PA without hormonal activity. There were no statistically significant differences between genotype distribution in patients with PA without hormonal activity and the control group (Table S2). Also, binary logistic regression of KDR rs2071559, rs1870377, and CFH rs1061170, rs1410996 in PA with and without hormonal activity showed no statistically significant results (Tables S3 and S4, respectively).

### 3.3 *KDR* rs2071559, rs1870377, and *CFH* rs1061170, rs1410996 associations with PAs invasiveness

After analyzing the distribution of genotypes and alleles of *KDR* rs2071559, rs1870377, and *CFH* rs1061170, rs1410996. *CFH* rs1061170 genotypes (TT, TC, CC) showed a statistically significant difference between non‐invasive PA and control group (47.2%, 52.8%, and 0.0% vs. 36.0%, 49.0%, and 15.0%, *p* = 0.039). Also, the T allele of this polymorphism was statistically significantly more frequent in patients with non‐invasive PA than in the control group (73.6 vs. 60.5, *p* = 0.034). Analysis of *KDR* rs2071559, rs1870377, and *CFH* rs1410996 showed no statistically significant results in non‐invasive PA patients and the control group (Table 4).

**TABLE 4:** Distribution of genotypes and alleles of *KDR* rs2071559, rs1870377 and *CFH* rs1061170, rs1410996 polymorphisms in patients with a non‐invasive PA and control group.

Polymorphisms	Non‐invasive PA, *N* (%)	Control group, *N* (%)	*p*‐value
*KDR* rs2071559			
AA	12 (33.3)	53 (26.5)	0.700
AG	16 (44.4)	98 (49.0)	
GG	8 (22.2)	49 (24.5)	
Total	36 (100)	200 (100)	
Allele			
A	40 (55.6)	204 (51.0)	0.476
G	32 (44.4)	196 (49.0)	
*KDR* rs1870377			
TT	21 (58.3)	108 (54.0)	0.700
TA	12 (33.3)	80 (40.0)	
AA	3 (8.3)	12 (6.0)	
Total	36 (100)	200 (100)	
Allele			
T	54 (75.0)	296 (74.0)	0.858
A	18 (25.0)	104 (26.0)	
*CFH* rs1061170			
TT	17 (47.2)	72 (36.0)	**0.039**
TC	19 (52.8)	98 (49.0)	
CC	0 (0.0)	30 (15.0)	
Total	36 (100)	200 (100)	
Allele			
T	53 (73.6)	242 (60.5)	**0.034**
C	19 (26.4)	158 (39.5)	
*CFH* rs1410996			
GG	9 (25.0)	72 (36.0)	0.387
GA	23 (63.9)	104 (52.0)	
AA	4 (11.1)	24 (12.0)	
Total	36 (100)	200 (100)	
Allele			
G	41 (56.9)	248 (62.0)	0.418
A	31 (43.1)	152 (38.0)	

Abbreviations: PA, pituitary adenoma; *p*‐value, significance level (differences considered significant when *p* < 0.05).

*Note:* Significant results are indicated in bold.

After evaluating the distribution of genotypes and alleles of *KDR* rs2071559, rs1870377, and *CFH* rs1061170, rs1410996 in patients with invasive PA and the control group, it was found that *KDR* rs2071559 both AA genotype and A allele were statistically significantly more frequent in patients with invasive PA group compared to the control group (42.2 vs. 26.5., *p* = 0.018; 63.3 vs. 51.0, *p* = 0.015, respectively). However, *KDR* rs1870377, and *CFH* rs1061170, rs1410996 showed no statistically significant results (Table 5).

**TABLE 5:** Distribution of genotypes and alleles of *KDR* rs2071559, rs1870377 and *CFH* rs1061170, rs1410996 polymorphisms in patients with an invasive PA and control group.

Polymorphism	Invasive PA, *N* (%)	Control group, *N* (%)	*p*‐value
*KDR* rs2071559			
AA	27 (42.2)^a^	53 (26.5)^a^	**0.047**
AG	27 (42.2)	98 (49.0)	
GG	10 (15.6)	49 (24.5)	
Total	64 (100)	200 (100)	
Allele			
A	81 (63.3)	204 (51.0)	**0.015**
G	47 (36.7)	196 (49.0)	
*KDR* rs1870377			
TT	32 (50.0)	108 (54.0)	0.407
TA	25 (39.1)	80 (40.0)	
AA	7 (10.9)	12 (6.0)	
Total	64 (100)	200 (100)	
Allele			
T	89 (69.5)	296 (74.0)	0.322
A	39 (30.5)	104 (26.0)	
*CFH* rs1061170			
TT	26 (40.6)	72 (36.0)	0.765
TC	30 (46.9)	98 (49.0)	
CC	8 (12.5)	30 (15.0)	
Total	64 (100)	200 (100)	
Allele			
T	82 (64.1)	242 (60.5)	0.471
C	46 (35.9)	158 (39.5)	
*CFH* rs1410996			
GG	24 (37.5)	72 (36.0)	0.845
GA	34 (53.1)	104 (52.0)	
AA	6 (9.4)	24 (12.0)	
Total	64 (100)	200 (100)	
Allele			
G	82 (64.1)	248 (62.0)	0.471
A	46 (35.9)	152 (38.0)	

Abbreviations: PA, pituitary adenoma; *p*‐value, significance level (differences considered significant when *p* < 0.05).

*Note:* Significant results are indicated in bold.

^a^ AA vs. AG+GG, *p* = 0.018.

After analyzing the influence of PA invasiveness on the disease manifestation, we found that the *KDR* rs1870377 was associated with 4.4‐ and 4.1‐fold increased odds of invasive PA development in codominant and recessive models (OR = 4.407; 95% CI: 1.157–16.785; *p* = 0.030; OR = 4.141; 95% CI: 1.159–14.802; *p* = 0.029, respectively) (Table 6). Moreover, binary logistic regression analysis of these SNPs in non‐invasive PA patients and the control group revealed no statistically significant results (Table S5).

**TABLE 6:** Binary logistic regression analysis of *KDR* rs2071559, rs1870377, and *CFH* rs1061170, rs1410996 in patients with invasive PA and control groups.

Model	Genotype/allele	OR (95% CI)	*p*‐value	AIC	
*KDR* rs2071559					
Co‐dominant	AG vs. GG	0.585 (0.240–1.426)	0.238	174.380	
	AA vs. GG	0.468 (0.152–1.440)	0.186		
Dominant	AG+AA vs. GG	0.547 (0.236–1.270)	0.161	172.558	
Recessive	AA vs. GG+AG	0.660 (0.249–1.749)	0.403	173.763	
Overdominant	AG vs. AA+GG	0.806 (0.372–1.748)	0.586	174.194	
Additive	A	0.672 (0.382–1.180)	0.166	172.535	
*KDR* rs1870377					
Co‐dominant	AT vs. TT	1.139 (0.497–2.611)	0.758	172.151	
	AA vs. TT	4.407 (1.157–16.785)	**0.030**		
Dominant	AT+AA vs. TT	1.413 (0.649–3.075)	0.384	173.727	
Recessive	AA vs. TT+AT	4.141 (1.159–14.802)	**0.029**	172.246	
Overdominant	AT vs. AA+TT	0.915 (0.419–2.000)	0.824	174.442	
Additive	A	1.662 (0.893–3.096)	0.109	171.937	
*CFH* rs1061170					
Co‐dominant	CT vs. TT	0.837 (0.358–1.953)	0.680	175.570	
	CC vs. TT	1.464 (0.358–1.953)	0.513		
Dominant	CT+CC vs. TT	0.961 (0.437–2.112)	0.920	174.481	
Recessive	CC vs. TT+CT	1.609 (0.562–4.607)	0.376	173.740	
Overdominant	CT vs. CC+TT	0.755 (0.346–1.649)	0.481	173.990	
Additive	C	1.114 (0.633–1.961)	0.708	174.351	
*CFH* rs1410996					
Co‐dominant	AG vs. GG	1.088 (0.474–2.496)	0.842	174.366	
	AA vs. GG	0.251 (0.050–1.249)	0.091		
Dominant	AG+AA vs. GG	0.876 (0.393–1.952)	0.746	174.387	
Recessive	AA vs. GG+AG	0.238 (0.052–1.097)	0.066	170.406	
Overdominant	AG vs. AA+GG	1.443 (0.660–3.154)	0.358	173.637	
Additive	A	0.693 (0.377–1.276)	0.239	173.073	

Abbreviations: AIC, akaike information criterion; OR, odds ratio; PA, pituitary adenoma; *p*‐value, significance level (differences considered significant when *p* < 0.05).

*Note:* Significant results are indicated in bold.

### 3.4 *KDR* rs2071559, rs1870377, and *CFH* rs1061170, rs1410996 associations with PAs recurrence

Analysis of the distribution of genotypes and alleles of *KDR* rs2071559, rs1870377, and *CFH* rs1061170, rs1410996 showed that *KDR* rs2071559 AA genotype and A allele were statistically significantly less frequent in the group of PA patients without recurrence compared to the control group (14.9 vs. 26.5, *p* = 0.043, 38.5 vs. 51.0, *p* = 0.009, respectively). While the GG genotype was statistically significantly more frequent in PA patients with no recurrence compared to control group individuals (37.8 vs. 24.5, *p* = 0.029) (Table 7). Binary logistic regression analysis revealed no statistically significant results of SNPs in PA without recurrence patients and control group (Table S6).

**TABLE 7:** Distribution of genotypes and alleles of *KDR* rs2071559, rs1870377, and *CFH* rs1061170, rs1410996 polymorphisms in the group of patients without PA recurrence and in the control group.

Polymorphism	PA without recurrence, *N* (%)	Control group, *N* (%)	*p*‐value
*KDR* rs2071559			
AA	11 (14.9)^a^	53 (26.5)^a^	0.097
AG	35 (47.3)	98 (49.0)	
GG	28 (37.8)^b^	49 (24.5)^b^	
Total	74 (100)	200 (100)	
Allele			
A	57 (38.5)	204 (51.0)	**0.009**
G	91 (61.5)	196 (49.0)	
*KDR* rs1870377			
TT	39 (52.7)	108 (54.0)	0.822
TA	29 (39.2)	80 (40.0)	
AA	6 (8.1)	12 (6.0)	
Total	74 (100)	200 (100)	
Allele			
T	107 (72.3)	296 (74.0)	0.688
A	41 (27.7)	104 (26.0)	
*CFH* rs1061170			
TT	31 (41.9)	72 (36.0)	0.292
TC	37 (50.0)	98 (49.0)	
CC	6 (8.1)	30 (15.0)	
Total	74 (100)	200 (100)	
Allele			
T	99 (66.9)	242 (60.5)	0.171
C	49 (33.1)	158 (39.5)	
*CFH* rs1410996			
GG	26 (35.1)	72 (36.0)	0.409
GA	43 (58.1)	104 (52.0)	
AA	5 (6.8)	24 (12.0)	
Total	74 (100)	200 (100)	
Allele			
G	95 (64.2)	248 (62.0)	0.638
A	53 (35.8)	152 (38.0)	

Abbreviations: PA, pituitary adenoma; *p*‐value, significance level (differences considered significant when *p* < 0.05).

*Note:* Significant results are indicated in bold.

^a^ AA vs. AG+GG *p* = 0.043.

^b^ GG vs. AA+AG *p* = 0.029.

Although the distribution of genotypes and alleles of *KDR* rs2071559, rs1870377, and *CFH* rs1061170, rs1410996 in the group of PA patients with recurrence and the control group showed no statistically significant results (Table S7). Binary logistic regression revealed that KDR rs1870377 was associated with 7.2‐ and 9.3‐fold increased odds of PA recurrence in codominant and recessive models, respectively (OR = 7.194; 95% CI: 1.366–37.892; *p* = 0.020; OR = 9.268; 95% CI: 1.854–46.321; *p* = 0.007) (Table 8).

**TABLE 8:** Binary logistic regression analysis of *KDR* rs2071559, rs1870377, and *CFH* rs1061170, rs1410996 in PA with recurrence and control groups.

Model	Genotype/allele	OR (95% CI)	*p*‐value	AIC
*KDR* rs2071559				
Co‐dominant	AG vs. GG	0.343 (0.091–1.291)	0.114	93.333
	AA vs. GG	0.460 (0.103–2.052)	0.308	
Dominant	AG+AA vs. GG	0.381 (0.113–1.281)	0.119	91.496
Recessive	AA vs. GG+AG	0.900 (0.253–3.193)	0.870	93.835
Overdominant	AG vs. AA+GG	0.502 (0.161–1.565)	0.235	92.407
Additive	A	0.649 (0.290–1.454)	0.294	92.729
*KDR* rs1870377				
Co‐dominant	AT vs. TT	0.524 (0.139–1.9790)	0.340	88.604
	AA vs. TT	7.194 (1.366–37.892)	**0.020**	
Dominant	AT+AA vs. TT	0.965 (0.320–2.912)	0.949	93.858
Recessive	AA vs. TT+AT	9.268 (1.854–46.321)	**0.007**	87.556
Overdominant	AT vs. AA+TT	0.390 (0.112–1.363)	0.140	91.469
Additive	A	1.649 (0.681–3.994)	0.268	92.646
*CFH* rs1061170				
Co‐dominant	CT vs. TT	0.975 (0.300–3.171)	0.967	95.842
	CC vs. TT	0.879 (0.147–5.253)	0.887	
Dominant	CT+CC vs. TT	0.954 (0.311–2.921)	0.934	93.855
Recessive	CC vs. TT+CT	0.890 (0.167–4.758)	0.892	93.844
Overdominant	CT vs. CC+TT	1.006 (0.333–3.036)	0.992	93.862
Additive	C	0.948 (0.420–2.142)	0.898	93.846
*CFH* rs1410996				
Co‐dominant	AG vs. GG	0.889 (0.267–2.957)	0.848	95.671
	AA vs. GG	0.672 (0.110–4.111)	0.667	
Dominant	AG+AA vs. GG	0.840 (0.265–2.662)	0.766	93.775
Recessive	AA vs. GG+AG	0.722 (0.138–3.788)	0.700	93.707
Overdominant	AG vs. AA+GG	0.996 (0.331–2.995)	0.995	93.862
Additive	A	0.838 (0.363–1.933)	0.679	93.689

Abbreviations: AIC, Akaike information criterion; OR, odds ratio; PA, pituitary adenoma; *p*‐value, significance level (differences considered significant when *p* < 0.05).

*Note:* Significant results are indicated in bold.

### 3.5 *KDR* rs2071559, rs1870377, and *CFH* rs1061170, rs1410996 haplotypes analysis

We performed a haplotype association analysis of *KDR* rs2071559, rs1870377, and *CFH* rs1061170, rs1410996 in patients with PA. Pairwise linkage disequilibrium between SNPs in PA patients is shown in Table 9.

**TABLE 9:** Linkage disequilibrium between studied polymorphisms in patients with PA.

SNPs	PA vs. controls		
	D′	*r* ^2^	*p*‐value
rs2071559‐rs1870377	0.253	0.020	<0.001
rs1061170‐rs1410996	1.000	0.365	0.0

Abbreviations: D′, the deviation between the expected haplotype frequency and the observed frequency; *r* ^2^, the square of the haplotype frequency correlation coefficient; SNP, single nucleotide polymorphism; *p*‐value, significance level when *p* < 0.05.

We analyzed haplotype frequencies, and statistical analysis of PA has shown that individuals carrying rs2071559, and rs1870377 haplotype A‐A were associated with 1.7‐fold increased odds of PA occurrence (OR = 1.67; 95% CI: 1.02–2.72; *p* = 0.041) (Table 10). Haplotype analysis of PA and *CFH* rs1061170 and rs1410996 showed no statistically significant results (Table S8).

**TABLE 10:** Haplotype association of *KDR* rs2071559 and rs1870377 with the predisposition to PA occurrence.

Haplotype	*KDR* rs2071559	*KDR* rs1870377	Frequency		OR (95% CI)	*p*‐value
			Control	PA		
1	G	T	0.382	0.333	1	–
2	A	T	0.358	0.382	1.19 (0.78–1.83)	0.42
3	A	A	0.176	0.223	1.67 (1.02–2.72)	**0.041**
4	G	A	0.108	0.062	0.63 (0.26–1.53)	0.31

Abbreviations: CI, confidence interval; OR, odds ratio; *p*‐value, significance level (statistically significant when *p* < 0.05).

*Note:* Significant results are indicated in bold.

### 3.6 KDR and CFH serum levels

**FIGURE 1:**
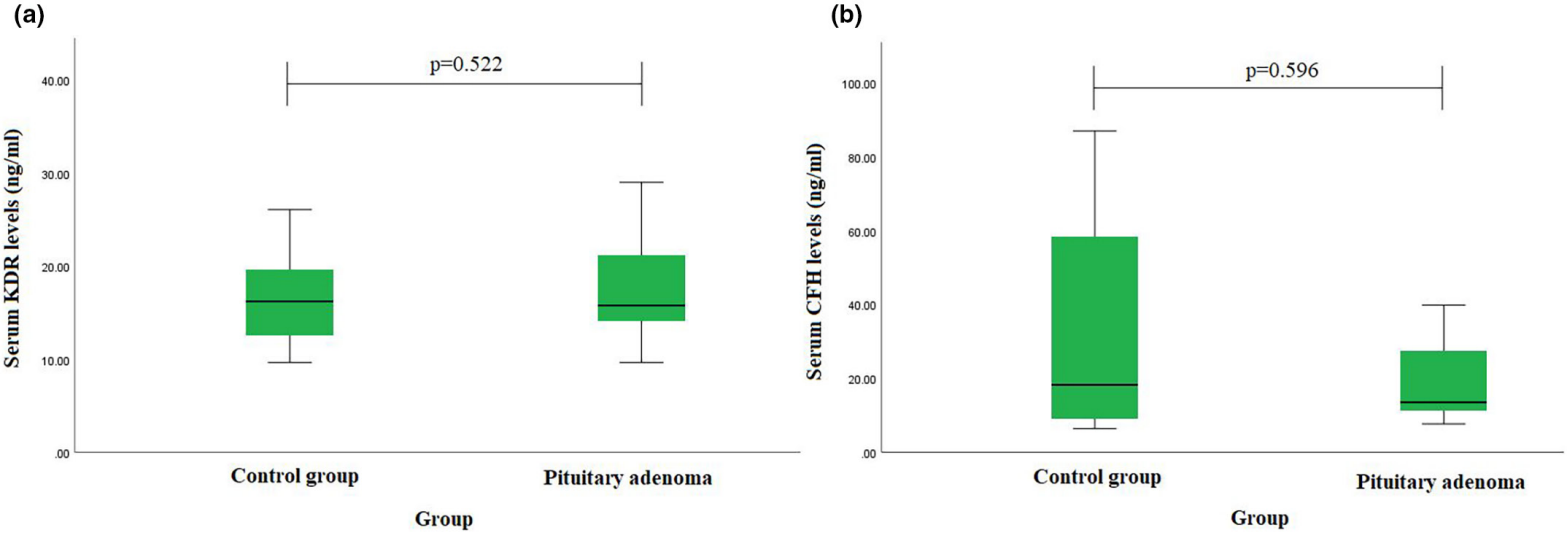
(a) Kinase insert domain receptor (KDR) and (b) complement factor H (CFH) concentrations in PA patients and healthy controls.

During the study, the concentration of KDR in the blood serum was examined in the groups of PA patients, and healthy individuals, but no statistically significant difference was found (median (IQR): 15.78 (8.19) vs. 16.21 (7.76), *p* = 0.522) (Figure 1a). Also, we evaluated CFH serum level, but no statistically significant differences were found (median (IQR): 18.23 (63.98) vs. 13.66 (24.43), *p* = 0.596) (Figure 1b).

## 4 DISCUSSION

PA tumorigenesis is not a fully understood process involving oncogene activation, tumor suppressor gene inactivation, and abnormal pituitary cell growth (Farrell & Clayton, 2000). PAs are sporadic in more than 95% of cases. Although whole genome sequencing studies have made significant progress in identifying their pathogenesis, the genetics of a significant fraction of pituitary tumors is still unclear (Melmed et al., 2022). The impact of *KDR* and *CFH* polymorphisms on the development of various tumors has been analyzed in many studies. To our knowledge, this is the first study targeting the association between *KDR* rs2071559, rs1870377, and *CFH* rs1061170, rs1410996 polymorphisms and PA occurrence.

As KDR regulates tumor angiogenesis, migration, and vascular permeability, it is associated with different types of cancers, including coronary heart disease, rheumatoid arthritis (RA) severity, age‐related macular degeneration (AMD), and recurrent miscarriage (Gao et al., 2016; Jinnin et al., 2008; Kaira et al., 2022; Lazzeri et al., 2012; Pădureanu et al., 2017; Paradowska‐Gorycka et al., 2019; Su et al., 2011; Vasconcelos et al., 2019; Wang et al., 2007). Pădureanu et al. have analyzed *KDR* rs2071559 polymorphism association with pancreatic cancer. The study showed that the GG genotype and G allele were more frequent among pancreatic cancer patients than controls (*p* = 0.036; *p* = 0.032, respectively), suggesting that the rs2071559 G allele may confer an increased risk of the disease (Pădureanu et al., 2017). According to a study done by Vasconcelos et al., inherited abnormalities in blood vessel formation related to *KDR* SNPs influence the risk and aggressiveness of high‐grade glioma (HGG) in an ethnically diverse population from Southeastern Brazil (Vasconcelos et al., 2019). Fraga with co‐authors investigated that carriers of the *KDR* rs2071559 A allele were more prone to higher VEGFR2/KDR expression in prostate epithelial cells (*p* = 0.006) (Fraga et al., 2017). Another study showed that higher KDR expression levels afford a very poor prognosis of colorectal cancer (Zhang et al., 2018). Also, the *KDR* rs2071559 G allele correlates with an increased risk of astrocytomas, while individuals with the A allele and genotype TA +AA of rs1870377 showed a protective effect against astrocytomas (Gao et al., 2016). Moreover, Qian et al. showed that the *KDR* rs2071559 AA genotype is associated with a fast baseline peritoneal solute transfer rate (PSTR), which is related to local membrane inflammation (*p* = 0.039) (Qian et al., 2022). As studies reveal controversial results, we observed that the *KDR* rs2071559 AA genotype and A allele are statistically significantly more frequent in PA patients than in the control group (*p* = 0.027; *p* = 0.028, respectively).

Complement factor H (CFH) is a primary regulator of the alternative complement pathway that controls C3 activation and modulates innate immune responses (Kopp et al., 2012). C3a stimulates anterior pituitary hormone release and activates the hypothalamic–pituitary–adrenal axis, a key regulator of inflammation. The complement molecules modulate systemic inflammatory responses through communication with the pituitary gland (Francis et al., 2003). However, CFH is associated with neurodegenerative diseases, decreased risk of recurrent pregnancy loss, periodontitis, myocardial infarction (MI), and a smoking‐related risk factor for lung cancer (Cho et al., 2019; Ezzeldin et al., 2015; Kardys et al., 2007; Maugeri et al., 2019; Salminen et al., 2022; Zhang et al., 2016). A study done by Zhang et al showed that *CFH* rs1061170 has strong associations with Alzheimer's disease (AD) in the European population and Han Chinese and European populations together (*p* < 0.001, *p*_meta_ = 0.0005, respectively) (Zhang et al., 2016). Li with co‐authors investigates the genetic relationship between *CFH* SNPs and susceptibility to sepsis caused by bacterial infections in Chinese Han populations. The frequency of the rs3753394 CT/TT genotype and T allele in *P. aeruginosa*‐induced septic patients was significantly higher than in the control group (*p* = 0.033’; *p* = 0.014, respectively) (Li et al., 2020). The *CFH* rs1061170 independently predicted mortality at discharge and 6 months and survival duration after spontaneous intracerebral hemorrhage (*p* = 0.019; *p* = 0.041, respectively) (Appelboom et al., 2011). It is conceivable that the *CFH* rs1061170 could be used to predict poor disease prognosis.

We found a correlation between *KDR* gene rs2071559 and PA hormonal activity, invasiveness, and recurrence. As far as we know, no studies analyzed the association between the *KDR* and *CFH* gene polymorphisms and PA invasiveness, hormonal activity, and recurrence. We revealed that the *KDR* polymorphism rs1870377 genotype had a 4.4‐ and 4.1‐fold increase in the odds of invasive PA development in codominant and recessive models (*p* = 0.030; *p* = 0.029, respectively). Also, The *KDR* rs1870377 polymorphism increases the odds of PA recurrence by 7.2‐ and 9.3‐fold in codominant and recessive models (*p* = 0.020; *p* = 0.007, respectively).

After analyzing the haplotype associations with pituitary adenoma, we revealed that individuals carrying *KDR* rs2071559, and rs1870377 haplotype A‐A had increased odds of PA occurrence. A study done by Gao et al. showed that haplotype C‐C‐T and C‐T‐T were linked to an increased risk of astrocytomas in the haplotype‐specific analysis. Variants of *KDR* rs2071559‐C and rs2305948‐T may increase the risk of astrocytomas, whereas mutants of *KDR* rs1870377‐A may reduce the risk of astrocytomas (Gao et al., 2016).

These results support the hypothesis that *KDR* and *CFH* genes are associated with inflammation in different organs, which can lead to an oncogenic process (Greten & Grivennikov, 2019). Further studies with larger sample sizes are warranted to evaluate *KDR*'s and *CFH*‘s predictive and prognostic value.

## 5 CONCLUSIONS

Results of the present study showed that *KDR* rs2071559 plays a crucial role in PA occurrence, hormonal activity, invasiveness, and recurrence. *KDR* rs1870377 increases the odds of PA recurrence and invasiveness. Also, *CFH* rs1061170 is associated with PA hormonal.

## AUTHOR CONTRIBUTIONS

Conceptualization, **Akvile Bruzaite**, **Greta Gedvilaite**, **Rasa Liutkeviciene**; methodology, **Akvile Bruzaite**, **Greta Gedvilaite**.; software, **Greta Gedvilaite**; validation, **Akvile Bruzaite**, **Greta Gedvilaite**; formal analysis, **Greta Gedvilaite**; investigation, **Akvile Bruzaite**; resources, **Rasa Liutkeviciene**, **Loresa Kriauciuniene**, data curation, **Greta Gedvilaite**; writing—original draft preparation, **Akvile Bruzaite**, **Greta Gedvilaite**, **Rasa Liutkeviciene**; writing—review and editing, **Akvile Bruzaite**, **Greta Gedvilaite**, **Rasa Liutkeviciene**; visualization, **Akvile Bruzaite**, **Greta Gedvilaite**, **Rasa Liutkeviciene**; supervision, **Rasa Liutkeviciene**; project administration, **Rasa Liutkeviciene**; funding acquisition, **Akvile Bruzaite**. All authors have read and agreed to the published version of the manuscript.

## FUNDING INFORMATION

This paper has received funding from the Research Council of Lithuania, agreement No. S‐SV‐22‐79.

## CONFLICT OF INTEREST STATEMENT

The authors declare no conflict of interest.

## ETHICS STATEMENT

Permission (No. BE‐2‐47; approved date: 25 December 2016) to perform the research was approved by the Ethics Committee for Biomedical Research at the Lithuanian University of Health Sciences (LUHS). All subjects have signed an agreement according to the Declaration of Helsinki. The study was conducted at the Laboratory of Ophthalmology, Neuroscience Institute, LUHS.

## INSTITUTIONAL REVIEW BOARD STATEMENT

The study was conducted in accordance with the Declaration of Helsinki, and approved by the Biomedical Research, Lithuanian University of Health Sciences (No. BE‐2‐47).

## INFORMED CONSENT STATEMENT

Informed consent was obtained from all subjects involved in the study.

## Supporting information


Table S1–S8.

## Data Availability

The data that support the findings of this study are available on request from the corresponding author. The data are not publicly available due to privacy or ethical restrictions.
