# Supplementary material for: Association of KDR (rs2071559, rs1870377), CFH (rs1061170, rs1410996) genes variants and serum levels with pituitary adenoma
Source: Mol Genet Genomic Med. 2023 Oct 6;12(1):e2289. doi: 10.1002/mgg3.2289 (PMC10767405; doi:10.1002/mgg3.2289)
Supplement: Supplementary file 1 — Table S1–S8. [file MGG3-12-e2289-s001.docx]

***Supplementary material***

**Table S1.**Binomial logistic regression analysis of *KDR* rs2071559, rs1870377, and *CFH* rs1061170, rs1410996 in PA patients and control groups.

| **Model** | **Genotype/allele** | **OR (95% CI)** | **p-Value** | **AIC** |
| --- | --- | --- | --- | --- |
| ***KDR* rs2071559** | | | | |
| Co-dominant | AG vs. GG  AA vs. GG | 0.585 (0.269-1.272)  0.676 (0.269-1.696) | 0.176  0.404 | 227.125 |
| Dominant | AG+AA vs. GG | 0.613 (0.297-1.264) | 0.185 | 225.235 |
| Recessive | AA vs. GG+AG | 0.943 (0.427-2.079) | 0.884 | 226.951 |
| Overdominant | AG vs. AA+GG | 0.695 (0.356-1.357) | 0.287 | 225.828 |
| Additive | A | 0.820 (0.501-1.282) | 0.356 | 226.115 |
| ***KDR* rs1870377** | | | | |
| Co-dominant | AT vs. TT  AA vs. TT | 1.071 (0.529-2.171)  3.227 (0.982-10.607) | 0.849  0.054 | 225.370 |
| Dominant | AT+AA vs. TT | 1.282 (0.658-2.495) | 0.465 | 226.436 |
| Recessive | AA vs. TT+AT | 3.126 (0.997-9.802) | 0.051 | 223.406 |
| Overdominant | AT vs. AA+TT | 0.907 (0.462-1.778) | 0.775 | 226.890 |
| Additive | A | 1.458 (0.860-2.473) | 0.162 | 225.020 |
| ***CFH* rs1061170** | | | | |
| Co-dominant | CT vs. TT  CC vs. TT | 1.029 (0.348-3.045)  1.022 (0.352-2.966) | 0.959  0.968 | 228.970 |
| Dominant | CT+CC vs. TT | 0.989 (0.503-1.944) | 0.974 | 226.971 |
| Recessive | CC vs. TT+CT | 0.975 (0.354-2.690) | 0.962 | 226.970 |
| Overdominant | CT vs. CC+TT | 1.000 (0.515-1.942) | 0.999 | 226.972 |
| Additive | C | 0.988 (0.601-1.623) | 0.962 | 226.970 |
| ***CFH* rs1410996** | | | | |
| Co-dominant | AG vs. GG  AA vs. GG | 1.014 (0.494-2.081)  0.345 (0.096-1.242) | 0.969  0.104 | 225.497 |
| Dominant | AG+AA vs. GG | 0.859 (0.428-1.724) | 0.669 | 226.789 |
| Recessive | AA vs. GG+AG | 0.342 (0.103-1.134) | 0.079 | 223.499 |
| Overdominant | AG vs. AA+GG | 1.294 (0.663-2.524) | 0.450 | 226.399 |
| Additive | A | 0.721 (0.425-1.223) | 0.225 | 225.471 |

PA – pituitary adenoma; OR: Odds ratio; AIC: Akaike information criterion; p-value – significance level (differences considered significant when p<0,05).

**Table S2.** Distribution of genotypes and alleles of *KDR* rs2071559, rs1870377, and *CFH* rs1061170, rs1410996 polymorphisms in the group of inactive PA patients and the control group.

| **Polymorphism** | **PA without hormonal activity, N (%)** | **Control group, N (%)** | **p-value** |
| --- | --- | --- | --- |
| ***KDR*rs2071559**  **AA**  **AG**  **GG**  **Total**  **Allele**  **A**  **G** | 16 (35,6)  20 (44,4)  9 (20,0)  45 (100)    52 (57,8)  38 (42,2) | 53 (26,5)  98 (49,0)  49 (24,5)  200 (100)    204 (51,0)  196 (49,0) | 0,463          0,245 |
| ***KDR*rs1870377**  **TT**  **TA**  **AA**  **Total**  **Allele**  **T**  **A** | 20 (44,4)  19 (42,2)  6 (13,3)  45 (100)    59 (65,6)  31 (34,4) | 108 (54,0)  80 (40,0)  12 (6,0)  200 (100)    296 (74,0)  104 (26,0) | 0,185          0,105 |
| ***CFH*rs1061170**  **TT**  **TC**  **CC**  **Total**  **Allele**  **T**  **C** | 17 (37,8)  22 (48,9)  6 (13,3)  45 (100)    56 (62,2)  34 (3,8) | 72 (36,0)  98 (49,0)  30 (15,0)  200 (100)    242 (60,5)  158 (39,5) | 0,951          0,762 |
| ***CFH*rs1410996**  **GG**  **GA**  **AA**  **Total**  **Allele**  **G**  **A** | 17 (37,8)  23 (51,1)  5 (11,1)  45 (100)    57 (63,3)  33 (36,7) | 72 (36,0)  104 (52,0)  24 (12,0)  200 (100)    248 (62,0)  152 (38,0) | 0,969          0,814 |

PA – pituitary adenoma; p-value – significance level (differences considered significant when p<0,05).

**Table S3.**Binomial logistic regression analysis of *KDR* rs2071559, rs1870377, and *CFH* rs1061170, rs1410996 in hormonal active PA patients and control groups.

| **Model** | **Genotype/allele** | **OR (95% CI)** | **p-Value** | **AIC** |
| --- | --- | --- | --- | --- |
| ***KDR* rs2071559** | | | | |
| Co-dominant | AG vs. GG  AA vs. GG | 0.565 (0.155-2.064)  0.462 (0.096-2.224) | 0.388  0.336 | 93.451 |
| Dominant | AG+AA vs. GG | 0.531 (0.156-1.802) | 0.310 | 91.530 |
| Recessive | AA vs. GG+AG | 0.673 (0.179-2.533) | 0.558 | 92.189 |
| Overdominant | AG vs. AA+GG | 0.812 (0.272-2.421) | 0.708 | 92.404 |
| Additive | A | 0.671 (0.303-1.487) | 0.326 | 91.559 |
| ***KDR* rs1870377** | | | | |
| Co-dominant | AT vs. TT  AA vs. TT | 0.882 (0.274-2.840)  3.398 (0.398-28.996) | 0.834  0.263 | 93.223 |
| Dominant | AT+AA vs. TT | 1.045 (0.346-3.156) | 0.938 | 92.538 |
| Recessive | AA vs. TT+AT | 3.606 (0.452-28.796) | 0.226 | 91.267 |
| Overdominant | AT vs. AA+TT | 0.940 (0.413-2.141) | 0.883 | 92.523 |
| Additive | A | 1.276 (0.500-3.253) | 0.610 | 92.285 |
| ***CFH* rs1061170** | | | | |
| Co-dominant | CT vs. TT  CC vs. TT | 0.943 (0.295-3.019)  0.880 (0.139-5.590) | 0.921  0.892 | 94.522 |
| Dominant | CT+CC vs. TT | 0.930 (0.309-2.802) | 0.897 | 92.528 |
| Recessive | CC vs. TT+CT | 0.906 (0.156-5.256) | 0.913 | 92.532 |
| Overdominant | CT vs. CC+TT | 0.968 (0.320-2.925) | 0.953 | 92.541 |
| Additive | C | 0.940 (0.413-2.141) | 0.883 | 92.523 |
| ***CFH* rs1410996** | | | | |
| Co-dominant | AG vs. GG  AA vs. GG | 1.007 (0.300-3.382)  0.271 (0.026-2.790) | 0.991  0.272 | 92.859 |
| Dominant | AG+AA vs. GG | 0.843 (0.257-2.767) | 0.778 | 92.466 |
| Recessive | AA vs. GG+AG | 0.270 (0.031-2.383) | 0.238 | 90.859 |
| Overdominant | AG vs. AA+GG | 1.364 (0.444-4.189) | 0.588 | 92.249 |
| Additive | A | 0.682 (0.276-1.683) | 0.406 | 91.835 |

PA – pituitary adenoma; OR: Odds ratio; AIC: Akaike information criterion; p-value – significance level (differences considered significant when p<0,05).

**Table S4.**Binomial logistic regression analysis of *KDR* rs2071559, rs1870377, and *CFH* rs1061170, rs1410996 in non-hormonal PA patients and control groups.

| **Model** | **Genotype/allele** | **OR (95% CI)** | **p-Value** | **AIC** |
| --- | --- | --- | --- | --- |
| ***KDR* rs2071559** | | | | |
| Co-dominant | AG vs. GG  AA vs. GG | 0.574 (0.238-1.387)  0.676 (0.239-1.908) | 0.218  0.459 | 178.038 |
| Dominant | AG+AA vs. GG | 0.606 (0.268-1.371) | 0.229 | 180.144 |
| Recessive | AA vs. GG+AG | 0.953 (0.388-2.337) | 0.916 | 181.547 |
| Overdominant | AG vs. AA+GG | 0.684 (0.319-1.466) | 0.329 | 180.592 |
| Additive | A | 0.799 (0.466-1.367) | 0.412 | 180.881 |
| ***KDR* rs1870377** | | | | |
| Co-dominant | AT vs. TT  AA vs. TT | 1.242 (0.555-2.779)  3.434 (0.960-12.288) | 0.598  0.058 | 180.244 |
| Dominant | AT+AA vs. TT | 1.480 (0.694-3.159) | 0.311 | 180.522 |
| Recessive | AA vs. TT+AT | 3.099 (0.922-10.413) | 0.067 | 178.522 |
| Overdominant | AT vs. AA+TT | 1.018 (0.476-2.174) | 0.964 | 181.556 |
| Additive | A | 1.607 (0.893-2.894) | 0.114 | 179.077 |
| ***CFH* rs1061170** | | | | |
| Co-dominant | CT vs. TT  CC vs. TT | 1.094 (0.484-2.471)  1.050 (0.311-3.541) | 0.829  0.938 | 183.511 |
| Dominant | CT+CC vs. TT | 1.085 (0.498-2.362) | 0.838 | 181.516 |
| Recessive | CC vs. TT+CT | 0.998 (0.325-3.066) | 0.997 | 181.558 |
| Overdominant | CT vs. CC+TT | 1.080 (0.509-2.293) | 0.840 | 181.517 |
| Additive | C | 1.043 (0.595-1.827) | 0.883 | 181.536 |
| ***CFH* rs1410996** | | | | |
| Co-dominant | AG vs. GG  AA vs. GG | 0.976 (0.435-2.189)  0.435 (0.109-1.740) | 0.954  0.239 | 181.851 |
| Dominant | AG+AA vs. GG | 0.847 (0.389-1.846) | 0.677 | 181.385 |
| Recessive | AA vs. GG+AG | 0.441 (0.120-1.622) | 0.218 | 179.855 |
| Overdominant | AG vs. AA+GG | 1.187 (0.558-2.527) | 0.656 | 181.359 |
| Additive | A | 0.758 (0.424-1.354) | 0.349 | 180.666 |

PA – pituitary adenoma; OR: Odds ratio; AIC: Akaike information criterion; p-value – significance level (differences considered significant when p<0,05).

**Table S5.**Binomial logistic regression analysis of *KDR* rs2071559, rs1870377, and *CFH* rs1061170, rs1410996 in non-invasive PA patients and control groups.

| **Model** | **Genotype/allele** | **OR (95% CI)** | **p-Value** | **AIC** |
| --- | --- | --- | --- | --- |
| ***KDR* rs2071559** | | | | |
| Co-dominant | AG vs. GG  AA vs. GG | 0.501 (0.148-1.696)  0.912 (0.241-3.446) | 0.267  0.892 | 108.655 |
| Dominant | AG+AA vs. GG | 0.625 (0.207-1.890) | 0.625 | 107.539 |
| Recessive | AA vs. GG+AG | 1.399 (0.455-4.300) | 0.558 | 107.879 |
| Overdominant | AG vs. AA+GG | 0.524 (0.187-1.474) | 0.221 | 106.674 |
| Additive | A | 0.945 (0.465-1.920) | 0.876 | 108.190 |
| ***KDR* rs1870377** | | | | |
| Co-dominant | AT vs. TT  AA vs. TT | 0.895 (0.308-2.597)  1.884 (0.279-12.711) | 0.838  0.515 | 109.695 |
| Dominant | AT+AA vs. TT | 0.998 (0.364-2.738) | 0.997 | 108.215 |
| Recessive | AA vs. TT+AT | 1.979 (0.310-12.628) | 0.470 | 107.737 |
| Overdominant | AT vs. AA+TT | 0.831 (0.296-2.330) | 0.724 | 108.090 |
| Additive | A | 1.125 (0.493-2.566) | 0.799 | 108.137 |
| ***CFH* rs1061170** | | | | |
| Co-dominant | CT vs. TT  CC vs. TT | 1.366 (0.485-3.848)  0.000 (0.000-0.000) | 0.555  0.998 | 105.540 |
| Dominant | CT+CC vs. TT | 1.074 (0.385-2.991) | 0.892 | 108.196 |
| Recessive | CC vs. TT+CT | 0.000 (0.000-0.000) | 0.998 | 103.893 |
| Overdominant | CT vs. CC+TT | 1.761 (0.634-4.892) | 0.278 | 107.017 |
| Additive | C | 0.773 (0.348-1.718) | 0.527 | 107.805 |
| ***CFH* rs1410996** | | | | |
| Co-dominant | AG vs. GG  AA vs. GG | 0.887 (0.285-2.760)  0.601 (0.104-3.469) | 0.836  0.569 | 109.872 |
| Dominant | AG+AA vs. GG | 0.825 (0.274-2.484) | 0.733 | 108.099 |
| Recessive | AA vs. GG+AG | 0.651 (0.134-3.168) | 0.595 | 107.915 |
| Overdominant | AG vs. AA+GG | 1.034 (0.370-2.888) | 0.950 | 108.211 |
| Additive | A | 0.805 (0.360-1.796) | 0.596 | 107.929 |

PA – pituitary adenoma; OR: Odds ratio; AIC: Akaike information criterion; p-value – significance level (differences considered significant when p<0,05).

**Table S6.**Binomial logistic regression analysis of *KDR* rs2071559, rs1870377, and *CFH* rs1061170, rs1410996 in PA without recurrence patients and control groups.

| **Model** | **Genotype/allele** | **OR (95% CI)** | **p-Value** | **AIC** |
| --- | --- | --- | --- | --- |
| ***KDR* rs2071559** | | | | |
| Co-dominant | AG vs. GG  AA vs. GG | 0.690 (0.291-1.638)  0.709 (0.246-2.039) | 0.401  0.523 | 186.905 |
| Dominant | AG+AA vs. GG | 0.696 (0.309-1.567) | 0.381 | 184.908 |
| Recessive | AA vs. GG+AG | 0.896 (0.359-2.233) | 0.813 | 185.607 |
| Overdominant | AG vs. AA+GG | 0.799 (0.379-1.686) | 0.556 | 185.316 |
| Additive | A | 0.827 (0.484-1.410) | 0.485 | 185.172 |
| ***KDR* rs1870377** | | | | |
| Co-dominant | AT vs. TT  AA vs. TT | 1.287 (0.592-2.796)  1.976 (0.448-8.724) | 0.524  0.369 | 186.707 |
| Dominant | AT+AA vs. TT | 1.358 (0.643-2.872) | 0.423 | 185.016 |
| Recessive | AA vs. TT+AT | 1.748 (0.418-7.306) | 0.444 | 185.113 |
| Overdominant | AT vs. AA+TT | 1.185 (0.561-2.501) | 0.656 | 185.465 |
| Additive | A | 1.348 (0.734-2.475) | 0.336 | 184.742 |
| ***CFH* rs1061170** | | | | |
| Co-dominant | CT vs. TT  CC vs. TT | 1.050 (0.474-2.325)  1.007 (0.294-3.456) | 0.905  0.991 | 187.648 |
| Dominant | CT+CC vs. TT | 1.041 (0.488-2.223) | 0.917 | 185.652 |
| Recessive | CC vs. TT+CT | 0.981 (0.309-3.112) | 0.974 | 185.662 |
| Overdominant | CT vs. CC+TT | 1.048 (0.498-2.206) | 0.902 | 185.648 |
| Additive | C | 1.018 (0.582-1.772) | 0.951 | 185.659 |
| ***CFH* rs1410996** | | | | |
| Co-dominant | AG vs. GG  AA vs. GG | 1.056 (0.472-2.362)  0.237 (0.048-1.185) | 0.895  0.080 | 183.213 |
| Dominant | AG+AA vs. GG | 0.864 (0.394-1.891) | 0.714 | 185.529 |
| Recessive | AA vs. GG+AG | 0.229 (0.050-1.050) | 0.058 | 181.230 |
| Overdominant | AG vs. AA+GG | 1.424 (0.670-3.031) | 0.358 | 184.812 |
| Additive | A | 0.681 (0.373-1.243) | 0.211 | 184.054 |

PA – pituitary adenoma; OR: Odds ratio; AIC: Akaike information criterion; p-value – significance level (differences considered significant when p<0,05).

**Table S7.**Distribution of genotypes and alleles of *KDR* rs2071559, rs1870377, and *CFH* rs1061170, rs1410996 polymorphisms in the group of PA patients with recurrence and in the control group.

| **Polymorphism** | **PA with recurrence, N (%)** | **Control group,**  **N (%)** | **p-value** |
| --- | --- | --- | --- |
| ***KDR*rs2071559**  **AA**  **AG**  **GG**  **Total**  **Allele**  **A**  **G** | 7 (26,9)  8 (30,8)  11 (42,3)  26 (100)    22 (42,3)  30 (57,7) | 53 (26,5)  98 (49,0)  49 (24,5)  200 (100)    204 (51,0)  196 (49,0) | 0,156          0,238 |
| ***KDR*rs1870377**  **TT**  **TA**  **AA**  **Total**  **Allele**  **T**  **A** | 14 (53,8)  8 (30,8)  4 (15,4)  26 (100)    36 (69,2)  16 (30,8) | 108 (54,0)  80 (40,0)  12 (6,0)  200 (100)    296 (74,0)  104 (26,0) | 0,156          0,464 |
| ***CFH*rs1061170**  **TT**  **TC**  **CC**  **Total**  **Allele**  **T**  **C** | 12 (46,2)  12 (46,2)  2 (7,7)  26 (100)    36 (69,2)  16 (30,8) | 72 (36,0)  98 (49,0)  30 (15,0)  200 (100)    242 (60,5)  158 (39,5) | 0,462          0,224 |
| ***CFH*rs1410996**  **GG**  **GA**  **AA**  **Total**  **Allele**  **G**  **A** | 7 (26,9)  14 (53,8)  5 (19,2)  26 (100)    28 (81,8)  24 (46,2) | 72 (36,0)  104 (52,0)  24 (12,0)  200 (100)    248 (62,0)  152 (38,0) | 0,474          0,257 |

PA – pituitary adenoma; p-value – significance level (differences considered significant when p<0,05).

**Table S8. Haplotype association of *CFH* rs1061170 and rs1410996** **with the predisposition to PA occurrence**.

| **Haplotype** | ***CFH*** rs1061170 | ***CFH*** rs1410996 | Frequency | | OR (95 % CI) | *p-*value |
| --- | --- | --- | --- | --- | --- | --- |
|  |  |  | Control | PA |  |  |
| 1 | T | A | 0.38 | 0.385 | 1.00 | - |
| 2 | C | G | 0.395 | 0.325 | 0.81 (0.53-1.24 | 0.33 |
| 3 | T | G | 0.225 | 0.29 | 1.28 (0.81-2.01) | 0.29 |

OR: odds ratio; CI: confidence interval; p-value: significance level (statistically significant when p < 0.05).
